# Molecular characterization of fluoroquinolone and/or cephalosporin resistance in *Shigella sonnei* isolates from yaks

**DOI:** 10.1186/s12917-018-1500-6

**Published:** 2018-06-07

**Authors:** Zhen Zhu, Yuxiang Shi, Xuzheng Zhou, Bing Li, Jiyu Zhang

**Affiliations:** 1 Key Laboratory of New Animal Drug Project of Gansu Province, Key Laboratory of Veterinary Pharmaceutical Development of the Ministry of Agriculture, Lanzhou Institute of Husbandry and Pharmaceutical Sciences of CAAS, Jiangouyan, Qilihe District, Lanzhou, 730050 China; 2 College of Life Science and Food Engineering, Hebei University of Engineering, Hanshan District, Handan, 056038 China

**Keywords:** *Shigella sonnei*, MLST, PFGE, Virulence gene, Antimicrobial resistance genes

## Abstract

**Background:**

Members of the genus *Shigella* are intestinal pathogens and a major cause of seasonal outbreaks of bacterial diarrhea worldwide. Although humans are the conventional hosts of *Shigella* species, expansion of the *Shigella* host range to certain animals was recently reported. To investigate the prevalence of *Shigella sonnei* (*S. sonnei*) in yaks and perform molecular characterization, we analyzed 1132 fresh yak diarrheal stool samples and collected a total of 44 S. sonnei isolates.

**Results:**

We performed multilocus sequence typing (MLST) and pulsed-field gel electrophoresis (PFGE) with *Xba*I-digested DNA to study genetic relatedness among the 44 isolates, which were differentiated into 4 sequence types (STs) and 32 PFGE types (PTs). All isolates harbored virulence genes, and 87.36% tested positive for invasion plasmid antigen H (*ipaH*), invasion associated locus (*ial*) and the *Shigella* enterotoxin gene *sen*. According to the results of antimicrobial susceptibility tests, 45.45% (20/44) were resistant to fluoroquinolones and/or cephalosporin. By sequencing the quinolone resistance determining region (QRDR) genes, we identified double mutations in *gyrA* (Ser83-Leu and Asp87-Asn) and a single mutation in *parC* (Ser80-Ile). All 12 fluoroquinolone-resistant *S. sonnei* isolates tested positive for the *aac(6′)-Ib-cr* gene but negative for *qepA*. Three isolates harbored *qnr* genes, including two with *qnrS* and one with *qnrB.* In addition, three types of β-lactamase genes, *bla*_*TEM-1*_, *bla*_*OXA-1*_ and *bla*_*CTX-M-14/79*_, were detected in cephalosporin-resistant isolates.

**Conclusions:**

The findings of this study have enriched our knowledge of fluoroquinolone- and/or cephalosporin-resistant *S. sonnei* isolates from yaks, which has important public health significance.

**Electronic supplementary material:**

The online version of this article (10.1186/s12917-018-1500-6) contains supplementary material, which is available to authorized users.

## Background

Bacteria of the genus *Shigella* are important members of the Enterobacteriaceae family and cause acute diarrhea in humans and animals [1–3]. This pathogen has been historically divided into 4 subgroups based on biochemical properties and group-specific O antigens in the outer membrane, specifically subgroups A (*S. dysenteriae*), B (*S. flexneri*), C (*S. boydii*) and D (*S. sonnei*). All four *Shigella* species cause shigellosis and are phylogenetically distinct from *Escherichia coli* [4, 5], with *S. flexneri* and *S. sonnei* being the most prevalent. Notably, *S. sonnei* has become the dominant subgroup in Asian countries in recent years [6, 7].

The specific pathogenicity of a given *Shigella* isolate is derived from the expression of diverse virulence genes that are associated with colonization, invasion/penetration and toxin-mediated disease [8]. The pathogenicity of these virulence genes is often multifactorial, and they are coordinately regulated [9]. An investigation of the virulence determinant genes in *Shigella* would help us better understand its pathogenicity. However, knowledge regarding the distribution of these genes in *Shigella* isolates from animals is limited.

For *Shigella* infections, prompt therapy with effective antimicrobial agents reduces the duration and severity of illness [10]. However, the progressive increase in resistance to commonly used antimicrobials, especially fluoroquinolones and extended-spectrum cephalosporins, among *Shigella* spp. poses a major therapeutic challenge to the control of diseases caused by these species [10, 11]. In addition, the emergence and global dissemination of multi-drug resistant (MDR) pathogens is increasing rapidly due to the unique ability of bacteria to acquire resistance factors (transmissible genes) from the environment or from other bacteria [12].

Foodborne pathogens are usually carried by the host animal and contaminate the food during the slaughtering process [13]. And food is the main route to human infection with Shigella, including antibiotic-resistant strains [14]. Foods implicated in human cases of shigellosis include fresh fruit and vegetables, raw oysters, deli meats, and unpasteurized milk [15]. Multidrug-resistant *Shigella* has been found in bovine-derived foods such as beef, milk and cheese, which predict potential threats to human [16].

The primary goal of this study was to investigate the incidence of *S. sonnei* in yaks with diarrheal disease in a plateau area. We analyzed the biochemical and serological characteristics, virulence gene profiles, antimicrobial resistance profiles, antimicrobial resistance genes (ARGs) and genotypes and genetic relatedness diversity of *S. sonnei* isolates.

## Results

### Identification of *S. sonnei* isolates

A total of 44 *S. sonnei* isolates were collected from 1132 fresh diarrheal stool samples from yaks in three Chinese provinces, Gansu, Qinghai and Tibet, from 2014 to 2016. Among these isolates, 24 (54.55%) were collected from Gansu, 15 (34.09%) were collected from Qinghai, and 5 (11.36%) were collected from Tibet. Based on the results of biochemical characterization assays, we observed that all 44 *S. sonnei* isolates possessed the typical biochemical features of *Shigella* species and demonstrated the ability to ferment I-D-galactopyranoside (ONPG), glucose (GLU), mannitol (MAN), melibiose (MEL) and arabinose (ARA). Although both serotypes were isolated in each region, phase I (33/44, 75%) was the predominant serotype, and the detection rate of *S. sonnei* phase I was three times higher than that of phase II (11/44, 25%).

### MLST-based genotype analysis

MLST was performed to analyze the genotypic diversity of *S. sonnei* isolates based on 15 housekeeping genes. The 44 isolates were divided into 4 sequence types (STs): ST76, ST116, ST125 and ST155. All four STs were previously reported and belonged to the same clonal complex (CC; CC29). The allele number for each locus and the ST designation are listed in Additional file 1: Table S1 and shared on the EcMLST website. Among these STs, ST116 (*n* = 27) and ST155 (*n* = 13) were the most common, accounting for 90.91% of all STs. ST116 encompassed 16 phase I and 11 phase II serotype isolates; furthermore, all phase II serotype *S. sonnei* isolates in the study were ST116. Interestingly, all four STs were detected in Gansu province, while only ST116 was observed in Qinghai and Tibet provinces (Table 1).

**Table 1:** Statistical analysis of the distribution of 4 MLST types among 44 *S. sonnei* isolates in different provinces

MLST types	Total (*n* = 44)		Gansu (*n* = 24)		Qinghai (*n* = 15)		Tibet (*n* = 5)	
	phase I (*n* = 33)	phase II (*n* = 11)	phase I (*n* = 20)	phase II (*n* = 4)	phase I (*n* = 10)	phase II (*n* = 5)	phase I (*n* = 3)	phase II (*n* = 2)
ST76	1 (3.03%)	0	1 (5%)	0	0	0	0	0
ST116	16 (48.48%)	11 (100%)	3 (15%)	4 (100%)	10 (100%)	5 (100%)	3 (100%)	2 (100%)
ST123	3 (9.09%)	0	3 (15%)	0	0	0	0	0
ST155	13 (39.39%)	0	13 (65%)	0	0	0	0	0

### PFGE-based genotype analysis

**Fig. 1:**
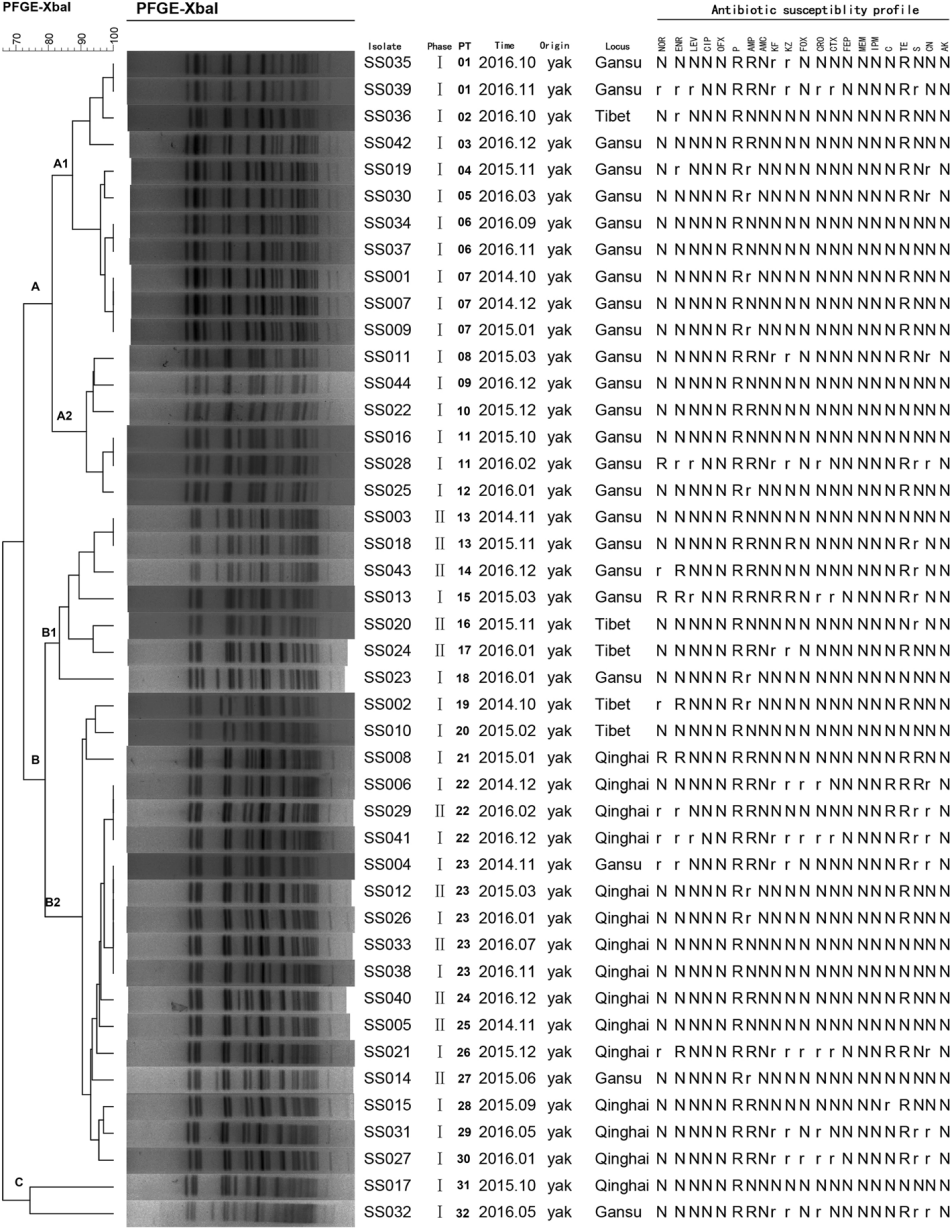
Dendrogram of 44 *Xba*I-digested *S. sonnei* isolates based on the cluster analysis of PFGE patterns. The dendrogram was constructed using the UPGMA clustering method. The corresponding antibiotic resistance profile, PFGE pattern and background information for each strain is listed on the right side of the dendrogram. R = resistance and no bacteriostatic ring; r = resistance and positive bacteriostatic ring; N = sensitive and intermediary bacteriostatic ring

The genotypes and genetic relatedness diversity of the 44 *S. sonnei* isolates were assessed by PFGE. At 80% similarity, *Xba*I-digested *S. sonnei* DNA generated 32 reproducible unique PFGE banding patterns (PT), yielding three major groups: A (*n* = 17), B (*n* = 25), and C (*n* = 2) (Fig. 1). Most isolates contained in PT group A were collected from the same geographical region (Gansu province) with the exception of one Tibet isolate (SS036). Group B contained the highest number of isolates, accounting for 56.82% of total isolates. In addition, almost all Qinghai isolates were clustered in group B2, with the exception of SS017. Interestingly, we also observed that different serotypes affect the PT cluster. In this study, all 17 group A isolates belonged to the phase I serotype, while all of the phase II isolates clustered into group B. The large number of PT types suggested a capricious genotypic and genetic diversity among *S. sonnei* strains, which adapt to different environments.

### Virulence genes

The virulence gene profiles of the isolates are listed in Additional file 2: Table S2. Only 3 virulence genes (*ipaH*, *ial* and *sen*) were observed in this study. Among them, the invasive plasmid gene *ipaH* was observed in all isolates, followed by *ial* (38/44, 86.36%) and *sen* (37/44, 84.09%). All isolates tested negative for *set1A*, *set1B* and *stx*.

According to their virulence gene distribution, the 44 *S. sonnei* isolates formed 3 gene profile patterns (Additional file 3: Table S3). VT3, which was positive for *ipaH*, *ial* and *sen*, contained the highest number of isolates (37, 87.36%) and was the major VT present in each province. VT2, which was positive for ipaH and ial, was only detected in one Qinghai isolate (SS005). Six isolates (13.64%), one in Gansu and five in Qinghai, belonged to VT1, which was only positive for *ipaH*.

### Antimicrobial susceptibility profiles

The antimicrobial resistance profiles of the 44 *S. sonnei* isolates to 21 antimicrobials are listed in Fig. 1 and Table 2. The antimicrobial susceptibility results showed that all *S. sonnei* isolates were sensitive to ciprofloxacin, ofloxacin, amoxicillin/clavulanic acid, cefepime, meropenem, imipenem, and amikacin but resistant to penicillin G. The resistance rates to other antimicrobials are as follows: ampicillin (31/44, 70.45%), tetracycline (30/44, 68.18%), cephazolin (14/44, 31.82%), streptomycin (14/44, 31.82%), cephalothin (13/44, 29.55%), enrofloxacin (12/44, 27.27%), gentamicin (12/44, 27.27%), norfloxacin (10/44, 22.73%), ceftriaxone (9/44, 20.45%), cefotaxime (5/44, 11.36%), levofloxacin (4/44, 9.09%), cefoxitin (4/44, 9.09%), and chloramphenicol (4/44, 9.09%). Although the observed resistance to most antimicrobials (with the exception of penicillin G, ampicillin and tetracycline) was less than 50%, multidrug-resistant (MDR) *S. sonnei* isolates, which were defined as being resistant to 3 or more antimicrobial agent subclasses, accounted for 43.18% of isolates (19/44).

**Table 2:** Statistical analysis of the results of antimicrobial susceptibility testing of 21 antibiotics for 44 *S. sonnei* isolates

Antimicrobial	Antimicrobial resistance rate [No. (%)]						
	Total (*n* = 44)	Gansu (*n* = 24)		Qinghai (*n* = 15)		Tibet (*n* = 5)	
		phase I (*n* = 20)	phase II (*n* = 4)	phase I (*n* = 10)	phase II (*n* = 5)	phase I (*n* = 3)	phase II (*n* = 2)
Norfloxacin	10 (22.73%)	4 (20%)	1 (25%)	3 (30%)	1 (20%)	1 (33.33%)	0
Enrofloxacin	12 (27.27%)	5 (25%)	1 (25%)	3 (30%)	1 (20%)	2 (66.67%)	0
Levofloxacin	4 (9.09%)	3 (15%)	0	1 (10%)	0	0	0
Ciprofloxacin	0	0	0	0	0	0	0
Ofloxacin	0	0	0	0	0	0	0
Penicillin G	44 (100%)	20 (100%)	4 (100%)	10 (100%)	5 (100%)	3 (100%)	2 (100%)
Ampicillin	31 (70.45%)	15 (75%)	3 (75%)	8 (80%)	3 (60%)	1 (33.33%)	1 (50%)
Amoxicillin/clavulanic acid	0	0	0	0	0	0	0
Cephalothin	13 (29.55%)	7 (35%)	0	5 (50%)	0	0	1 (50%)
Cephazolin	14 (31.82%)	7 (35%)	1 (25%)	5 (50%)	0	0	1 (50%)
Cefoxitin	4 (9.09%)	0	0	4 (40%)	0	0	0
Ceftriaxone	9 (20.45%)	4 (20%)	0	5 (50%)	0	0	0
Cefotaxime	5 (11.36%)	2 (10%)	0	3 (30%)	0	0	0
Cefepime	0	0	0	0	0	0	0
Meropenem	0	0	0	0	0	0	0
Imipenem	0	0	0	0	0	0	0
Chloramphenicol	4 (9.09%)	0	0	3 (30%)	1 (20%)	0	0
Tetracycline	30 (68.18%)	14 (70%)	2 (50%)	8 (80%)	3 (60%)	2 (66.67%)	1 (50%)
Streptomycin	14 (31.82%)	5 (25%)	2 (50%)	5 (50%)	1 (20%)	0	1 (50%)
Gentamicin	12 (27.27%)	6 (30%)	0	5 (50%)	1 (20%)	0	0
Amikacin	0	0	0	0	0	0	0

Remarkably, the rates of resistance to fluoroquinolones and cephalosporin were 27.275% (12/44) and 31.82% (14/44), respectively, and 13.64% (6/44) of isolates were resistant to both fluoroquinolones and cephalosporin. Moreover, all fluoroquinolone-resistant isolates belonged to the MDR group. Interestingly, the fluoroquinolone- and/or cephalosporin-resistant isolates demonstrated diverse antimicrobial resistance profiles, and no isolates had the same resistance profile (Table 3 and Fig. 1).

**Table 3:** Analysis of amino acid types in QRDRs and ARGs in *S. sonnei* isolates with resistance to fluoroquinolones and/or cephalosporin

Strain name	Antimicrobial resistance profiles	Fluoroquinolone resistance genes					Cephalosporin resistance genes		
		QRDR			PMQR genes		*TEM*	*OXA*	*CTX-M*
		*gyrA*		*parC*	*aac(6′)-Ib-cr*	*qnr*			
		83 (S)	87 (D)	80 (S)					
SS036	ENR/TE	L	D	S	*aac(6′)-Ib-cr*	NA	---	---	---
SS019	ENR/AMP/TE/CN	L	D	S	*aac(6′)-Ib-cr*	NA	---	---	---
SS002	NOR/ENR/AMP/TE	L	D	S	*aac(6′)-Ib-cr*	NA	---	---	---
SS008	NOR/ENR/AMP/TE/S	L	N	S	*aac(6′)-Ib-cr*	qnrB	---	---	---
SS043	NOR/ENR/AMP/TE/S	L	D	I	*aac(6′)-Ib-cr*	NA	---	---	---
SS029	NOR/ENR/AMP/C/TE/S/CN	L	N	S	*aac(6′)-Ib-cr*	NA	---	---	---
SS018	AMP/KZ/TE/S	---	---	---	---	---	*TEM-1*	NA	NA
SS024	AMP/KF/KZ/TE	---	---	---	---	---	*TEM-1*	NA	NA
SS035	AMP/KF/KZ/TE	---	---	---	---	---	*TEM-1*	NA	NA
SS011	AMP/KF/KZ/TE/CN	---	---	---	---	---	*TEM-1*	NA	NA
SS031	AMP/KF/KZ/CRO/TE/S/CN	---	---	---	---	---	*TEM-1*	*OXA-1*	NA
SS032	AMP/KF/KZ/CRO/TE/S/CN	---	---	---	---	---	*TEM-1*	*OXA-1*	NA
SS006	AMP/KF/KZ/FOX/CRO/C/TE/S/CN	---	---	---	---	---	*TEM-1*	*OXA-1*	NA
SS027	AMP/KF/KZ/FOX/CRO/CTX/TE/S/CN	---	---	---	---	---	*TEM-1*	NA	*CTX-M-79*
SS004	NOR/ENR/AMP/KF/KZ/TE/S/CN	L	N	S	*aac(6′)-Ib-cr*	NA	*TEM-1*	*OXA-1*	NA
SS028	NOR/ENR/LEV/AMP/KF/KZ/CRO/TE/S/CN	L	N	I	*aac(6′)-Ib-cr*	NA	*TEM-1*	*OXA-1*	NA
SS013	NOR/ENR/LEV/AMP/KF/KZ/CRO/CTX/TE/S	L	N	I	*aac(6′)-Ib-cr*	NA	*TEM-1*	*OXA-1*	*CTX-M-14*
SS039	NOR/ENR/LEV/AMP/KF/KZ/CRO/CTX/TE/S	L	N	I	*aac(6′)-Ib-cr*	NA	*TEM-1*	*OXA-1*	*CTX-M-14*
SS021	NOR/ENR/AMP/KF/KZ/FOX/CRO/CTX/C/TE/CN	L	N	S	*aac(6′)-Ib-cr*	*qnrS*	*TEM-1*	*OXA-1*	*CTX-M-14*
SS041	NOR/ENR/LEV/AMP/KF/KZ/FOX/CRO/CTX/TE/S/CN	L	N	I	*aac(6′)-Ib-cr*	*qnrS*	*TEM-1*	*OXA-1*	*CTX-M-14*

*S* Ser, *L* Leu, *D* Asp, *N* Asn, *I* Ile, *NA* not available; “---”: not detected

### Molecular analysis of antimicrobial resistance determinants

In the present study, all four QRDR genes (*gyrA*, *gyrB*, *parC*, and *parE*) were sequenced and compared with reference sequences. According to the results, double mutations in *gyrA* (Ser83-Leu and Asp87-Asn) and a single mutation in *parC* (Ser80-Ile) were associated with fluoroquinolone resistance in *S. sonnei* isolates (Table 3). The encoded Ser83-Leu amino acid substitution in *gyrA* was observed in every fluoroquinolone-resistant isolate, and 66.67% (8/12) of fluoroquinolone-resistant isolates possessed the Asp87-Asn mutation in *gyrA*. Only four isolates harbored all three amino acid substitutions. Interestingly, all four of these isolates belonged to the phase I serotype.

In addition to the identification of substitutions in QRDR genes, PMQR genes were amplified and sequenced. According to the results, all fluoroquinolone-resistant isolates harbored the *aac(6′)-Ib-cr* gene. The *qepA* gene, which encodes an efflux pump, was not observed in the studied isolates. Only three Qinghai isolates tested positive for the *qnr* gene, with two having *qnrS* and one having *qnrB* (Table 3).

For the 14 cephalosporin-resistant isolates, only three β-lactamase gene types (*bla*_*TEM*_, *bla*_*OXA*_ and *bla*_*CTX-M*_) were observed. All isolates tested positive for the *bla*_*TEM*_ gene and showed 100% identity with the *bla*_*TEM − 1*_ subtype. And all the third-cephalosporin resistant isolates were positive for *bla*_*OXA*_ (*bla*_*OXA-1*_), except SS027. Five out of the fourteen cephalosporin-resistant isolates tested positive for *bla*_*CTX-M*_, 4 of which had the *bla*_*CTX-M-14*_ gene, and 1 had the *bla*_*CTX-M-79*_ gene.

## Discussion

Bacillary dysentery caused by *Shigella* is endemic throughout the world. Humans are the natural hosts of *Shigella*, although reports of *Shigella* infections in animals (fish, calves, piglets, and chickens) have emerged [2, 17–19]. We previously confirmed the existence of diverse *S. sonnei* isolates in yak in local epidemiological studies. The rate of *S. sonnei* isolation from the diarrhea of yaks was 3.89% (44/1132), and the predominant serotype was phase I, which was isolated three times more frequently than phase II. In addition, 54 *S. flexneri* isolates with diverse serotypes were reported in our previous study [19].

MLST and PFGE have shown comparable discrimination in terms of their ability to subtype *S. sonnei*. In this study, 44 *S. sonnei* isolates from yaks were grouped into 4 STs based on 15 housekeeping genes. Comparison of these genes with the standard gene types on the EcMLST website revealed that the different STs of these isolates were primarily attributable to three genes, *aspC*, *mutS*, and *ropS*. In addition, all isolates belonged to the same clonal complex, suggesting a relatively close genetic relationship among them. In our study, the majority of isolates (*n* = 27 and *n* = 13) belonged to ST116 and ST155, respectively. However, ST76, ST116, and ST114 were previously reported in human isolates [20, 21]. PFGE is a broadly applicable typing method that has high resolution for the molecular characterization of several enteric bacteria, including *Shigella* [20]. Based on our PFGE analysis, the *S. sonnei* isolates in the present study were heterogeneous and distributed into 32 PTs. The clustering of these diverse PTs allows us to learn more about the epidemiological characteristics of *S. sonnei* in specific geographical regions. PFGE appeared to have a higher discriminatory power for genotypes and genetic relatedness diversity analysis than MLST.

Virulence genes are responsible for the invasion of virulent *Shigella* strains into intestinal epithelial cells, resulting in dysentery in hosts [22]. *ipaH*, which is typically used as a marker of *Shigella*, was harbored by all isolates. Additionally, in this study, most isolates simultaneously harbored the *ial* and *sen* genes. The diversity of the observed virulence genes suggested the isolates were pathogenic. Moreover, virulence genes were frequently located in megaplasmids, which allow *Shigella* to acquire and disseminate bacterial invasion genes through mobile genetic elements [23].

Recently, antimicrobial-resistant and MDR *S. sonnei* isolates have been widely isolated [20, 23]. In this study, we isolated MDR *S. sonnei* isolates from yaks. More than half of the isolates were resistant to conventional and commonly used antimicrobials: penicillin G (100%), ampicillin (70.45%), and tetracycline (68.18%). Fortunately, for most antimicrobials, the rates and levels of resistance of *Shigella* isolates from yaks were less than those from isolates from livestock [24]. Notably, although only low rates of resistance and MIC values to fluoroquinolones and/or cephalosporin were observed, some *S. sonnei* had acquired the ability to survive the selective pressures of these antimicrobials, which will limit the control and treatment of shigellosis.

Fluoroquinolone resistance is primarily mediated by the accumulation of sequential mutations in four QRDR genes: *gyrA* and *gyrB*, which encode DNA gyrase, and *parC* and *parE*, which encode and topoisomerase IV [25]. The primary mutations are located in *gyrA* and *parC*. According to a previous study, the major mutations in *gyrA* codons 81 (Gly → Ser), 83 (Ser → Leu), 87 (Asp→Asn/Gly/Tyr), and 211 (His→Tyr) and *parC* codons 80 (Ser → Ile), 83 (Ser → Leu), and 129 (Ser → Pro) are associated with quinolone and/or fluoroquinolone resistance in *Shigella* [11, 24, 26, 27]. Furthermore, the continuous accumulation of mutations at multiple positions and different mutations at the same position may lead to diverse levels of resistance [23]. In this study, we identified QRDR mutations in all fluoroquinolone-resistant *S. sonnei*, observing only a few classic amino acid substitutions in *gyrA* and *parC*.

The PMQR determinants are typically located on mobile or transposable genetic elements, which may facilitate dissemination among *Shigella* species and other members of the Enterobacteriaceae family [28, 29]. Recently, the diversity of PMQR determinants has emerged as an important issue worldwide [27]. Among the PMQR genes, *aac(6′)-Ib-cr*, which encodes aminoglycoside acetyl transferase and is responsible for reduced fluoroquinolone activity, was the most prevalent in our study. The *qnr*-encoded family of proteins protects DNA gyrase from quinolones and confers low-level resistance [30]. *QnrS* were majority prevalence in *Shigella* (*S.sonnei* and *S.flexneri*) in China, while *qnrB* previously in India [23, 26]. In addition, the plasmid-mediated efflux pump-encoding gene *qepA* was detected at lower frequencies [27]. The presence of PMQR genes may help microorganisms develop resistance by conferring mutations and facilitating the selection of higher-level quinolone resistance [31].

ESBLs continue to be the major defense mechanism against cephalosporins among Gram-negative bacteria [32]. Since first being identified in the 1980s, different types of ESBLs belonging to the *TEM*, *OXA*, *SHV* and *CTX-M* families have been widely reported in Enterobacteriaceae, including *Shigella* species [33, 34]. The global spread of ESBL-producing *Shigella* is difficult to control and eradicate because these bacteria reside in the intestines of humans and animals and harbor epidemic resistance plasmids. Therefore, the prevalence of ESBL-producing *S. sonnei* in yaks represents a substantial threat.

## Conclusion

Antimicrobial-resistant *Shigella* remains a serious threat to humans and animals; however, knowledge of the molecular epidemiology of this pathogen in yaks is limited. *Shigella* species typically harbor various plasmids that are associated with invasion into host intestinal epithelial cells and antimicrobial resistance and may be transferred between different bacteria. In the present study the *S. sonnei* strains isolated from yaks that tested positive for diverse virulence genes and ARGs represent a potential threat to hosts, including humans. Therefore, continuous and extensive surveillance will be necessary to control and reduce the threat of foodborne pathogens, such as *Shigella*, *Salmonella* and *E. coli*.

## Methods

### Bacterial isolates and serological confirmation

A total of 44 *S. sonnei* isolates were obtained from yaks with diarrhea from October 2014 to December 2016. All yaks have hardly been exposed to any antibiotic except for a few of penicillin to against the disease. All isolates were collected directly from fresh stool samples, plated on Salmonella-Shigella (SS) selective agar and confirmed on MacConkey (MAC) agar at 37 °C for 18 to 24 h. Colorless, semitransparent, smooth, and moist circular plaques were considered presumptive *Shigella* isolates and were selected for further confirmation [34]. The presumptive positive *Shigella* isolates were identified using API 20 E kits (bioMerieux, Marcy-l’Etoile, France), and serotypes were tested with a commercially available kit (Denka Seiken; Tokyo, Japan) according to the manufacturer’s recommendations.

### Multilocus sequence typing (MLST)

MLST was performed for each isolate according to the protocols described on the EcMLST website (http://www.shigatox.net/ecmlst). The following PCR amplification conditions were used for 15 housekeeping genes: 94 °C for 5 min, followed by 30 cycles of 94 °C for 30 s, 55 °C for 30 s, and 72 °C for 1 min, with incubation for 10 min at 72 °C with ExTaq DNA polymerase (Takara; Dalian, China). The PCR products were sequenced bi-directionally, and sequences of the 15 housekeeping genes were edited using SeqMan 7.0. Finally, the sequences were uploaded to the EcMLST website for comparison, which allowed us to determine the gene and ST type [35].

### Pulsed-field gel electrophoresis (PFGE)

All *S. sonnei* isolates were analyzed for genetic relatedness by PFGE after digestion with the restriction enzyme *Xba*I (TaKaRa; Japan) according to the recommendations of a previous study [36]. *Salmonella enterica* serotype Braenderup strain H9812 was used as a molecular size maker. Electrophoresis was performed with a Bio-Rad CHEF Mapper XA system (Bio-Rad; USA) in a 1% SeaKem Gold agarose gel (Lonza; USA) in 0.5X TBE buffer with a size range of 30–700 kb. The PFGE run conditions were 6 V/cm with a switch from 2.16 to 54.17 s for 21 h at 14 °C.

After gel electrophoresis, gels were stained with ethidium bromide, rinsed, and photographed with a Universal Hood II (Bio-Rad; USA). The PFGE patterns were analyzed with BioNumerics using the Dice similarity coefficient, unweighted pair-group method with arithmetic mean (UPGMA) and 1.0% tolerance.

### Detection of virulence genes by PCR

All *S. sonnei* isolates were examined for the presence of six virulence genes, invasion plasmid antigen H (*ipaH*), invasion associated locus (*ial*), Shiga toxin gene (*stx*), and *Shigella* enterotoxin genes (*set1A*, *set1B*, and *sen*), by PCR according to published procedures [23, 37]. The primers for these virulence genes are listed in Additional file 4: Table S4. Amplification products were separated by 1% agarose gel electrophoresis and stained with ethidium bromide.

### Antimicrobial susceptibility

Antimicrobial susceptibility testing was performed by the Kirby-Bauer disc diffusion method on Muller-Hinton agar (MHA) plates following the guidelines of the Clinical and Laboratory Standards Institute (CLSI) using commercially available antimicrobial discs (Oxoid, UK). Twenty-one antimicrobial discs were used in this study: norfloxacin (NOR, 10 μg), enrofloxacin (ENR, 5 μg), levofloxacin (LEV, 5 μg), ciprofloxacin (CIP, 5 μg), ofloxacin (OFX, 5 μg), penicillin G (P, 10 μg), ampicillin (AMP, 10 μg), amoxicillin/clavulanic acid (AMC, 30 μg), cephalothin (KF, 30 μg), cephazolin (KZ, 30 μg), cefoxitin (FOX, 30 μg), ceftriaxone (CRO, 30 μg), cefotaxime (CTX, 30 μg), cefepime (FEP, 30 μg), imipenem (IPM, 10 μg), meropenem (MEM, 10 μg), chloramphenicol (C, 30 μg), tetracycline (TE, 30 μg), streptomycin (S, 10 μg), gentamicin (CN, 10 μg), and amikacin (AK, 30 μg). *E. coli* ATCC 25922 was used as a control strain. The standard of antibiotic susceptibility for CLSI were list in Additional file 5: Table S5.

### Detection of β-lactamase and quinolone resistance genes

Cephalosporin- and/or fluoroquinolone-resistant *S. sonnei* isolates were assayed by PCR using 24 primer panels to determine the underlying mechanism conferring resistance (Additional file 6: Table S6). Specifically, 6 plasmid-mediated quinolone resistance (PMQRs) determinant genes (*qnrA*, *qnrB*, *qnrD*, *qnrS*, *qepA* and *aac(6′)-Ib-cr*) and 4 quinolone resistance determining region (QRDR) genes (*gyrA*, *gyrB*, *parC* and *parE*) were amplified to clarify the underlying mechanism conferring resistance to quinolones [38–40]. Extended-spectrum β-lactamase (ESBL) genes (*bla*_*CTX-M*_, *bla*_*SHV*_, *bla*_*TEM*_, and *bla*_*OXA*_) were detected in cephalosporin-resistant isolates [39, 41, 42].

## Additional files

Additional file 1: **Table S1.** MLST allelic profiles and ST designations of 44 *S. sonnei* isolates from this study. (DOCX 45 kb)

Additional file 2: **Table S2.** Statistical analysis of the presence of virulence genes in each S. sonnei isolate. (DOCX 39 kb)

Additional file 3: **Table S3.** Statistical analysis of the occurrence of each virulence gene profile in different provinces. (DOCX 36 kb)

Additional file 4: **Table S4.** Primers used to detect virulence genes. (DOCX 39 kb)

Additional file 5: **Table S5.** The standard of antibiotic susceptibility for K-B disc-diffusion method. (DOCX 59 kb)

Additional file 6: **Table S6.** Primers used to detect antibiotic resistance determinant genes. (DOCX 81 kb)

## Abbreviations

ESBL: Extended-spectrum β-lactamase
MLST: Multilocus sequence typing
PFGE: Pulsed-field gel electrophoresis
PMQRs: Plasmid-mediated quinolone resistance
QRDR: Quinolone resistance determining region
VT: Virulence gene profile types
