# Supplementary material for: Molecular characterization of fluoroquinolone and/or cephalosporin resistance in Shigella sonnei isolates from yaks
Source: BMC Vet Res. 2018 Jun 7;14:177. doi: 10.1186/s12917-018-1500-6 (PMC5992640; doi:10.1186/s12917-018-1500-6)
Supplement: Supplementary file 1 — Table S1. MLST allelic profiles and ST designations of 44 S. sonnei isolates from this study. (DOCX 45 kb) [file 12917_2018_1500_MOESM1_ESM.docx]

**Table S1.** **MLST allelic profiles and ST designations of 44 *S. sonnei* isolates from this study.**

| **Isolates name** | **Allele profiles** | | | | | | | | | | | | | | | **ST** |
| --- | --- | --- | --- | --- | --- | --- | --- | --- | --- | --- | --- | --- | --- | --- | --- | --- |
|  | ***arcA*** | ***aroE*** | ***aspC*** | ***clpX*** | ***cyaA*** | ***dnaG*** | ***fadD*** | ***grpE*** | ***icdA*** | ***lysP*** | ***mdh*** | ***mtlD*** | ***mutS*** | ***rpoS*** | ***uidA*** |  |
| SS001 | 9 | 13 | 4 | 19 | 13 | 3 | 18 | 3 | 21 | 14 | 23 | 13 | 40 | 17 | 1 | 155 |
| SS002 | 9 | 13 | 18 | 19 | 13 | 3 | 18 | 3 | 21 | 14 | 23 | 13 | 17 | 68 | 1 | 116 |
| SS003 | 9 | 13 | 18 | 19 | 13 | 3 | 18 | 3 | 21 | 14 | 23 | 13 | 17 | 68 | 1 | 116 |
| SS004 | 9 | 13 | 18 | 19 | 13 | 3 | 18 | 3 | 21 | 14 | 23 | 13 | 17 | 68 | 1 | 116 |
| SS005 | 9 | 13 | 18 | 19 | 13 | 3 | 18 | 3 | 21 | 14 | 23 | 13 | 17 | 68 | 1 | 116 |
| SS006 | 9 | 13 | 18 | 19 | 13 | 3 | 18 | 3 | 21 | 14 | 23 | 13 | 17 | 68 | 1 | 116 |
| SS007 | 9 | 13 | 4 | 19 | 13 | 3 | 18 | 3 | 21 | 14 | 23 | 13 | 40 | 17 | 1 | 155 |
| SS008 | 9 | 13 | 18 | 19 | 13 | 3 | 18 | 3 | 21 | 14 | 23 | 13 | 17 | 68 | 1 | 116 |
| SS009 | 9 | 13 | 4 | 19 | 13 | 3 | 18 | 3 | 21 | 14 | 23 | 13 | 40 | 17 | 1 | 155 |
| SS010 | 9 | 13 | 18 | 19 | 13 | 3 | 18 | 3 | 21 | 14 | 23 | 13 | 17 | 68 | 1 | 116 |
| SS011 | 9 | 13 | 4 | 19 | 13 | 3 | 18 | 3 | 21 | 14 | 23 | 13 | 17 | 68 | 1 | 123 |
| SS012 | 9 | 13 | 18 | 19 | 13 | 3 | 18 | 3 | 21 | 14 | 23 | 13 | 17 | 68 | 1 | 116 |
| SS013 | 9 | 13 | 18 | 19 | 13 | 3 | 18 | 3 | 21 | 14 | 23 | 13 | 17 | 68 | 1 | 116 |
| SS014 | 9 | 13 | 18 | 19 | 13 | 3 | 18 | 3 | 21 | 14 | 23 | 13 | 17 | 68 | 1 | 116 |
| SS015 | 9 | 13 | 18 | 19 | 13 | 3 | 18 | 3 | 21 | 14 | 23 | 13 | 17 | 68 | 1 | 116 |
| SS016 | 9 | 13 | 4 | 19 | 13 | 3 | 18 | 3 | 21 | 14 | 23 | 13 | 40 | 17 | 1 | 155 |
| SS017 | 9 | 13 | 18 | 19 | 13 | 3 | 18 | 3 | 21 | 14 | 23 | 13 | 17 | 68 | 1 | 116 |
| SS018 | 9 | 13 | 18 | 19 | 13 | 3 | 18 | 3 | 21 | 14 | 23 | 13 | 17 | 68 | 1 | 116 |
| SS019 | 9 | 13 | 4 | 19 | 13 | 3 | 18 | 3 | 21 | 14 | 23 | 13 | 40 | 17 | 1 | 155 |
| SS020 | 9 | 13 | 18 | 19 | 13 | 3 | 18 | 3 | 21 | 14 | 23 | 13 | 17 | 68 | 1 | 116 |
| SS021 | 9 | 13 | 18 | 19 | 13 | 3 | 18 | 3 | 21 | 14 | 23 | 13 | 17 | 68 | 1 | 116 |
| SS022 | 9 | 13 | 4 | 19 | 13 | 3 | 18 | 3 | 21 | 14 | 23 | 13 | 17 | 68 | 1 | 123 |
| SS023 | 9 | 13 | 18 | 19 | 13 | 3 | 18 | 3 | 21 | 14 | 23 | 13 | 17 | 68 | 1 | 116 |
| SS024 | 9 | 13 | 18 | 19 | 13 | 3 | 18 | 3 | 21 | 14 | 23 | 13 | 17 | 68 | 1 | 116 |
| SS025 | 9 | 13 | 4 | 19 | 13 | 3 | 18 | 3 | 21 | 14 | 23 | 13 | 40 | 17 | 1 | 155 |
| SS026 | 9 | 13 | 18 | 19 | 13 | 3 | 18 | 3 | 21 | 14 | 23 | 13 | 17 | 68 | 1 | 116 |
| SS027 | 9 | 13 | 18 | 19 | 13 | 3 | 18 | 3 | 21 | 14 | 23 | 13 | 17 | 68 | 1 | 116 |
| SS028 | 9 | 13 | 4 | 19 | 13 | 3 | 18 | 3 | 21 | 14 | 23 | 13 | 40 | 17 | 1 | 155 |
| SS029 | 9 | 13 | 18 | 19 | 13 | 3 | 18 | 3 | 21 | 14 | 23 | 13 | 17 | 68 | 1 | 116 |
| SS030 | 9 | 13 | 4 | 19 | 13 | 3 | 18 | 3 | 21 | 14 | 23 | 13 | 40 | 17 | 1 | 155 |
| SS031 | 9 | 13 | 18 | 19 | 13 | 3 | 18 | 3 | 21 | 14 | 23 | 13 | 17 | 68 | 1 | 116 |
| SS032 | 9 | 13 | 4 | 19 | 13 | 3 | 18 | 3 | 21 | 14 | 23 | 13 | 17 | 17 | 1 | 76 |
| SS033 | 9 | 13 | 18 | 19 | 13 | 3 | 18 | 3 | 21 | 14 | 23 | 13 | 17 | 68 | 1 | 116 |
| SS034 | 9 | 13 | 4 | 19 | 13 | 3 | 18 | 3 | 21 | 14 | 23 | 13 | 40 | 17 | 1 | 155 |
| SS035 | 9 | 13 | 4 | 19 | 13 | 3 | 18 | 3 | 21 | 14 | 23 | 13 | 40 | 17 | 1 | 155 |
| SS036 | 9 | 13 | 18 | 19 | 13 | 3 | 18 | 3 | 21 | 14 | 23 | 13 | 17 | 68 | 1 | 116 |
| SS037 | 9 | 13 | 4 | 19 | 13 | 3 | 18 | 3 | 21 | 14 | 23 | 13 | 40 | 17 | 1 | 155 |
| SS038 | 9 | 13 | 18 | 19 | 13 | 3 | 18 | 3 | 21 | 14 | 23 | 13 | 17 | 68 | 1 | 116 |
| SS039 | 9 | 13 | 4 | 19 | 13 | 3 | 18 | 3 | 21 | 14 | 23 | 13 | 40 | 17 | 1 | 155 |
| SS040 | 9 | 13 | 18 | 19 | 13 | 3 | 18 | 3 | 21 | 14 | 23 | 13 | 17 | 68 | 1 | 116 |
| SS041 | 9 | 13 | 18 | 19 | 13 | 3 | 18 | 3 | 21 | 14 | 23 | 13 | 17 | 68 | 1 | 116 |
| SS042 | 9 | 13 | 4 | 19 | 13 | 3 | 18 | 3 | 21 | 14 | 23 | 13 | 40 | 17 | 1 | 155 |
| SS043 | 9 | 13 | 18 | 19 | 13 | 3 | 18 | 3 | 21 | 14 | 23 | 13 | 17 | 68 | 1 | 116 |
| SS044 | 9 | 13 | 4 | 19 | 13 | 3 | 18 | 3 | 21 | 14 | 23 | 13 | 17 | 68 | 1 | 123 |
