# Supplementary material for: Molecular characterization of fluoroquinolone and/or cephalosporin resistance in Shigella sonnei isolates from yaks
Source: BMC Vet Res. 2018 Jun 7;14:177. doi: 10.1186/s12917-018-1500-6 (PMC5992640; doi:10.1186/s12917-018-1500-6)
Supplement: Supplementary file 2 — Table S2. Statistical analysis of the presence of virulence genes in each S. sonnei isolate. (DOCX 39 kb) [file 12917_2018_1500_MOESM2_ESM.docx]

**Table S2. Statistical analysis of the presence of virulence genes in each *S. sonnei* isolate.**

| **Isolate name** | **Virulence gene** **profiles** | | | | | | VT |
| --- | --- | --- | --- | --- | --- | --- | --- |
|  | *ipaH* | *ial* | *sen* | *Set1A* | *Set1B* | *stx* |  |
| SS001 | + | + | + | - | - | - | 3 |
| SS002 | + | + | + | - | - | - | 3 |
| SS003 | + | + | + | - | - | - | 3 |
| SS004 | + | - | - | - | - | - | 1 |
| SS005 | + | + | - | - | - | - | 2 |
| SS006 | + | - | - | - | - | - | 1 |
| SS007 | + | + | + | - | - | - | 3 |
| SS008 | + | + | + | - | - | - | 3 |
| SS009 | + | + | + | - | - | - | 3 |
| SS010 | + | + | + | - | - | - | 3 |
| SS011 | + | + | + | - | - | - | 3 |
| SS012 | + | + | + | - | - | - | 3 |
| SS013 | + | + | + | - | - | - | 3 |
| SS014 | + | + | + | - | - | - | 3 |
| SS015 | + | - | - | - | - | - | 1 |
| SS016 | + | + | + | - | - | - | 3 |
| SS017 | + | + | + | - | - | - | 3 |
| SS018 | + | + | + | - | - | - | 3 |
| SS019 | + | + | + | - | - | - | 3 |
| SS020 | + | + | + | - | - | - | 3 |
| SS021 | + | + | + | - | - | - | 3 |
| SS022 | + | + | + | - | - | - | 3 |
| SS023 | + | + | + | - | - | - | 3 |
| SS024 | + | + | + | - | - | - | 3 |
| SS025 | + | + | + | - | - | - | 3 |
| SS026 | + | - | - | - | - | - | 1 |
| SS027 | + | - | - | - | - | - | 1 |
| SS028 | + | + | + | - | - | - | 3 |
| SS029 | + | - | - | - | - | - | 1 |
| SS030 | + | + | + | - | - | - | 3 |
| SS031 | + | + | + | - | - | - | 3 |
| SS032 | + | + | + | - | - | - | 3 |
| SS033 | + | + | + | - | - | - | 3 |
| SS034 | + | + | + | - | - | - | 3 |
| SS035 | + | + | + | - | - | - | 3 |
| SS036 | + | + | + | - | - | - | 3 |
| SS037 | + | + | + | - | - | - | 3 |
| SS038 | + | + | + | - | - | - | 3 |
| SS039 | + | + | + | - | - | - | 3 |
| SS040 | + | + | + | - | - | - | 3 |
| SS041 | + | + | + | - | - | - | 3 |
| SS042 | + | + | + | - | - | - | 3 |
| SS043 | + | + | + | - | - | - | 3 |
| SS044 | + | + | + | - | - | - | 3 |
| Total | 44 | 38 | 37 | 0 | 0 | 0 | --- |

+: positive; -: negative ; VT: virulence gene profile types.

Total: The number of positive isolates.
