# Supplementary material for: Molecular characterization of fluoroquinolone and/or cephalosporin resistance in Shigella sonnei isolates from yaks
Source: BMC Vet Res. 2018 Jun 7;14:177. doi: 10.1186/s12917-018-1500-6 (PMC5992640; doi:10.1186/s12917-018-1500-6)
Supplement: Supplementary file 3 — Table S3. Statistical analysis of the occurrence of each virulence gene profile in different provinces. (DOCX 36 kb) [file 12917_2018_1500_MOESM3_ESM.docx]

**Table S3. Statistical analysis of the occurrence of each virulence gene profile in different provinces.**

| VT | Virulence gene profiles | No. of isolates (%) | | | |
| --- | --- | --- | --- | --- | --- |
|  |  | Total (n=44) | Gansu (n=24) | Qinghai (n=15) | Tibet (n=5) |
| 1 | *ipaH* | 6 (13.64%) | 1 (4.17%) | 5 (33.33%) | 0 |
| 2 | *ipaH+ial* | 1 (2.27%) | 0 | 1 (6.67%) | 0 |
| 3 | *ipaH+ial+sen* | 37 (86.36%) | 23 (95.83%) | 9 (60.00%) | 5 (100%) |

VT: virulence gene profile type.
