# Supplementary material for: Molecular characterization of fluoroquinolone and/or cephalosporin resistance in Shigella sonnei isolates from yaks
Source: BMC Vet Res. 2018 Jun 7;14:177. doi: 10.1186/s12917-018-1500-6 (PMC5992640; doi:10.1186/s12917-018-1500-6)
Supplement: Supplementary file 4 — Table S4. Primers used to detect virulence genes. (DOCX 39 kb) [file 12917_2018_1500_MOESM4_ESM.docx]

**Table S4. Primers used to detect virulence genes.**

| Target | Primer sequence (5’ to 3’) | (bp) | Amplicon size (bp) |
| --- | --- | --- | --- |
| *ipaH* | F: TGGAAAAACTCAGTGCCTCT | 1063–1083 | 423 |
|  | R: CCAGTCCGTAAATTCATTCT | 1466–1485 |  |
| *ial* | F: CTGGATGGTATGGTGAGG | 5340–5357 | 320 |
|  | R: GGAGGCCAACAATTATTTCC | 5640–5659 |  |
| *sen* | F: ATGTGCCTGCTATTATTTAT | 380–399 | 799 |
|  | R: CATAATAATAAGCGGTCAGC | 1158–1178 |  |
| *Set1A* | F: TCACGCTACCATCAAAGA | 460–477 | 309 |
|  | R: TATCCCCCTTTGGTGGTA | 751–768 |  |
| *Set1B* | F: GTGAACCTGCTGCCGATATC | 70–89 | 147 |
|  | R: ATTAGTGGATAAAAATGACG | 197–216 |  |
| *stx* | F: ACCCTGTAACGAAGTTTGCG | --- | 140 |
|  | R: CATCTCATGCGACTACTTGAC | --- |  |
