# Supplementary material for: Molecular characterization of fluoroquinolone and/or cephalosporin resistance in Shigella sonnei isolates from yaks
Source: BMC Vet Res. 2018 Jun 7;14:177. doi: 10.1186/s12917-018-1500-6 (PMC5992640; doi:10.1186/s12917-018-1500-6)
Supplement: Supplementary file 5 — Table S5. The standard of antibiotic susceptibility for K-B disc-diffusion method. (DOCX 59 kb) [file 12917_2018_1500_MOESM5_ESM.docx]

**Table S5** **The standard of antibiotic susceptibility for K-B disc-diffusion method.**

| Antibiotics | | Dose | Breakpoint of MIC (mm) | | |
| --- | --- | --- | --- | --- | --- |
|  |  |  | S | I | R |
| Ciprofloxacin | CIP | 5 | ≥21 | 16-20 | ≤15 |
| Enrofloxacin | ENR | 5 | ≥21 | 17-20 | ≤16 |
| Norfloxacin | NOR | 10 | ≥17 | 13-16 | ≤12 |
| Ofloxacin | OFX | 5 | ≥16 | 13-15 | ≤12 |
| Levofloxacin | LEV | 5 | ≥17 | 14-18 | ≤13 |
| Penicillin G | P | 10 | --- | --- | --- |
| Ampicillin | AMP | 10 | ≥17 | 14-16 | ≤13 |
| Amoxycillin/clavulanic acid | AMC | 20/10 | ≥18 | 14-17 | ≤14 |
| Cephalothin | KF | 30 | ≥18 | 15-17 | ≤14 |
| Cephazolin | KZ | 30 | ≥23 | 20-22 | ≤19 |
| Cefoxitin | FOX | 30 | ≥18 | 15-17 | ≤14 |
| Ceftriaxone | CRO | 30 | ≥26 | 23-25 | ≤22 |
| Cefotaxime | CTX | 30 | ≥23 | 20-22 | ≤19 |
| Cefepime | FEP | 30 | ≥18 | 15-17 | ≤14 |
| Imipenem | IPM | 10 | ≥23 | 20-22 | ≤19 |
| Meropenem | MEM | 10 | ≥23 | 20-22 | ≤19 |
| Chloramphenicol | C | 30 | ≥18 | 13-17 | ≤12 |
| Tetracycline | TE | 30 | ≥15 | 12-14 | ≤11 |
| Streptomycin | S | 10 | ≥15 | 12-14 | ≤11 |
| Gentamicin | CN | 10 | ≥15 | 13-14 | ≤12 |
| Amikacin | AK | 30 | ≥17 | 15-16 | ≤14 |

S: sensitive; I: intermediary; R: resistance.
