# Supplementary material for: Molecular characterization of fluoroquinolone and/or cephalosporin resistance in Shigella sonnei isolates from yaks
Source: BMC Vet Res. 2018 Jun 7;14:177. doi: 10.1186/s12917-018-1500-6 (PMC5992640; doi:10.1186/s12917-018-1500-6)
Supplement: Supplementary file 6 — Table S6. Primers used to detect antibiotic resistance determinant genes. (DOCX 81 kb) [file 12917_2018_1500_MOESM6_ESM.docx]

**Table S6. Primers used to detect antibiotic resistance determinant genes.**

| Target | Primer sequence (5’ to 3’) | Amplicon size (bp) | Reference |
| --- | --- | --- | --- |
| **PMQRs** | | | |
| *qnrA* | F: ATTTCTCACGCCAGGATTTG | 516 | Colobatiu et al. (2015) |
|  | R: GATCGGCAAAGGTTAGGTCA |  |  |
| *qnrB* | F: GATCGTGAAAGCCAGAAAGG | 476 | Colobatiu et al. (2015) |
|  | R: ACGATGCCTGGTAGTTGTCC |  |  |
| *qnrD* | F: CGAGATCAATTTACGGGGAATA | 656 | Tariq et al. (2012) |
|  | R: AACAAGCTGAAGCGCCTG |  |  |
| *qnrS* | F: ACGACATTCGTCAACTGCAA | 417 | Colobatiu et al. (2015) |
|  | R: TAAATTGGCACCCTGTAGGC |  |  |
| *aac(6′)-Ib-cr* | F: CCCGCTTTCTCGTAGCA | 544 | Colobatiu et al. (2015) |
|  | R: TTAGGCATCACTGCGTCTTC |  |  |
| *qepA* | F: CGTGTTGCTGGAGTTCTTC | 403 | Colobatiu et al. (2015) |
|  | R: CTGCAGGTACTGCGTCATG |  |  |
| **QRDR** | | | |
| *gyrA* | F: TACACCGGTCAACATTGAGG | 648 | Hu et al. (2007) |
|  | R: TTAATGATTGCCGCCGTCGG |  |  |
| *gyrB* | F: TGAAATGACCCGCCGTAAAGG | 309 | Hu et al. (2007) |
|  | R: GCTGTGATAACGCAGTTTGTCCGGG |  |  |
| *parC* | F: GTACGTGATCATGGACCGTG | 531 | Hu et al. (2007) |
|  | R: TTCGGCTGGTCGATTAATGC |  |  |
| *parE* | F: ATGCGTGCGGCTAAAAAAGTG | 290 | Hu et al. (2007) |
|  | R: TCGTCGCTGTCAGGATCGATAC |  |  |
| **β-lactamase** | | | |
| *bla_CTX-M-1_* | F: GGTTAAAAAATCACTGCGTC | 873 | Matar et al. (2007) |
|  | R: TTACAAACCGTCGGTGACGA |  |  |
| *bla_CTX-M-2_* | F: CGACGCTACCCCTGCTATT | 552 | Zong et al. (2008) |
|  | R: CCAGCGTCAGATTTTTCAGG |  |  |
| *bla_CTX-M-8_* | F: TCGCGTTAAGCGGATGATGC | 689 | Zong et al. (2008) |
|  | R: AACCCACGATGTGGGTAGC |  |  |
| *bla_CTX-M-9_* | F: AGAGTGCAACGGATGATG | 868 | Matar et al. (2007) |
|  | R: CCAGTTACAGCCCTTCGG |  |  |
| *bla_CTX-M-25_* | F: TTGTTGAGTCAGCGGGTTGA | 497 | Liu et al. (2015) |
|  | R: GCGCGACCTTCCGGCCAAAT |  |  |
| *bla_SHV_* | F: CGCCGGGTTATTCTTATTTGTCGC | 1015 | Zong et al. (2008) |
|  | R: TCTTTCCGATGCCGCCGCCAGTCA |  |  |
| *bla_TEM_* | F: ATGAGTATTCAACATTTCCG | 1080 | Tariq et al. (2012) |
|  | R: CCAATGCTTAATCAGTGAGG |  |  |
| *bla_OXA_* | F: ATTAAGCCCTTTACCAAACCA | 890 | Matar et al. (2007) |
|  | R: AAGGGTTGGGCGATTTTGCCA |  |  |
